# High Stroma T-Cell Infiltration is Associated with Better Survival in Stage pT1 Bladder Cancer

**DOI:** 10.3390/ijms21218407

**Published:** 2020-11-09

**Authors:** Sabine Hülsen, Eleonora Lippolis, Fulvia Ferrazzi, Wolfgang Otto, Luitpold Distel, Rainer Fietkau, Stefan Denzinger, Johannes Breyer, Maximilian Burger, Simone Bertz, Markus Eckstein, Annette Ebner, Arndt Hartmann, Carol-I. Geppert

**Affiliations:** 1 Institute of Pathology, Friedrich-Alexander-University, Erlangen-Nürnberg, 91054 Erlangen, Germany; sabine.huelsen@outlook.com (S.H.); Fulvia.Ferrazzi@uk-erlangen.de (F.F.); Simone.Bertz@uk-erlangen.de (S.B.); Markus.Eckstein@uk-erlangen.de (M.E.); a_ebner@gmx.de (A.E.); Arndt.Hartmann@uk-erlangen.de (A.H.); 2 Institute of Human Genetics, Friedrich-Alexander-University, Erlangen-Nürnberg, 91054 Erlangen, Germany; eleonora.lippolis19@gmail.com; 3 Department of Nephropathology, Institute of Pathology, Friedrich-Alexander-University, Erlangen-Nürnberg, 91054 Erlangen, Germany; 4 Caritas St. Josef Medical Center, Department of Urology, Regensburg University, 93053 Regensburg, Germany; Wolfgang.Otto@klinik.uni-regensburg.de (W.O.); sdenzinger@caritasstjosef.de (S.D.); jbreyer@caritasstjosef.de (J.B.); mburger@caritasstjosef.de (M.B.); 5 Department of Radiation Therapy, Friedrich-Alexander-University, Erlangen-Nürnberg, 91054 Erlangen, Germany; Luitpold.Distel@uk-erlangen.de (L.D.); Rainer.Fietkau@uk-erlangen.de (R.F.)

**Keywords:** pT1 bladder cancer, immunoscore, tumor-infiltrating lymphocytes (TILs), CD3^+^ lymphocytes, CD8^+^ lymphocytes, progression, survival

## Abstract

Stage pT1 bladder cancer (BC) shows highly diverse outcomes. Predictive markers are required to stratify patients for personalized treatment. The present study aimed to validate immune response quantification as a prognostic marker. Patients with pT1 BC (*n* = 167) treated by transurethral resection of the bladder (TURB) were enrolled. Formaldehyde-fixed paraffin-embedded material was stained for CD3 and CD8. Corresponding T cells were counted in three regions with the highest immune response. Numbers of tertiary lymphoid structures (TLS) and lymphocyte aggregates (LA) were quantified. High CD3^+^ stroma T-cell infiltration was associated with improved survival (*p* = 0.045), especially in the G3 subgroup (*p* = 0.01). Cluster with higher immune response showed less recurrence (*p* = 0.034) and favorable overall survival (OS) (*p* = 0.019). In contrast, higher CD3^+^ and CD8^+^ tumor T-cell infiltration seemed to have a negative impact on prognosis. TLS and LA were more frequently observed in G3 tumors, indicating an increased anti-tumoral immune response. We proved the role of immune cell infiltration and showed that higher infiltration numbers of CD3^+^ (not CD8^+^) lymphocytes in the stroma are associated with favorable outcome. Immune cell quantification could be used as a marker to help stratify patients’ risk and therefore, to optimize patients’ management and follow-up examination as well as possible therapies.

## 1. Introduction

With an incidence of approximately 550,000 worldwide in 2018 [1], bladder cancer (BC) is one of the most challenging cancer entities in urology, especially when it initially appears as non-muscle-invasive bladder cancer (NMIBC). Although this disease is not as advanced as muscle-invasive bladder cancer (MIBC), it may involve aggressive tumors with a high tendency to progression [2]. It is difficult to predict patient outcomes especially in stage T1G3 bladder cancer, which invades the subepithelial tissue, but not the deeper muscle layer. Shahin et al. described “the rule of 30 s”—although 30% of T1G3 BC do not relapse, 30% progress to muscle-invasive cancer and 30% of patients die due to advanced stage disease [3].

While there is an opportunity for an organ-preserving approach by resection and adjuvant bacillus Calmette–Guérin (BCG) instillations after initial transurethral resection of the bladder (TURB), there are also recommendations for early cystectomy in patients with T1G3 bladder cancer, especially in patients with adverse clinicopathological features [4]. 

The idea of establishing an immunoscore for clinical practice was first described in 2010 [5]. Latest studies elaborated an immunoscore for colorectal cancer [6]. Other investigations suggested an immunoscore for hepatocellular cancer [7] or gastric cancer [8]. The numbers of two different lymphocyte subtypes, CD3^+^ (= all T cells) and CD8^+^ T cells (= cytotoxic subset), in two different areas, invasive margin and tumor core were proposed to be most informative in colorectal cancer. A high immune response results in a higher immunoscore and is associated with a longer OS and disease-free survival (DFS) [9].

For MIBC, Pfannstiel et al. have already performed a comprehensive analysis addressing the fundamental importance of the tumor microenvironment in combination with immunoscoring [10]. In contrast, studies showed that for early invasive stage T1 BC, not all NMIBC risk factors are feasible to determine prognosis [11,12]. New histopathological and molecular markers are urgently needed to select patients who are at higher risk for worse outcome and could therefore profit from early cystectomy and to identify those who would be overtreated [13].

In this study, we aimed to identify patient subsets with T1 bladder cancer who may benefit from more precise treatment or closer follow-up examinations. Marker expression levels (CD3^+^ and CD8^+^ T cells) were correlated with clinical outcome in T1 BC patients, who did not undergo early cystectomy. 

## 2. Results

### 2.1. T-Cell Infiltration Patterns in Stroma and Tumor

Immune cell infiltration was successfully quantified with the applied time-effective semiautomatic method. Quantification for stroma and tumor was possible for CD3^+^ T cells and for CD8^+^ T cells separately. For CD3^+^ T-cell immune response, the mean value for stroma infiltration was 2839 cells/mm^2^ (0–8940). For tumor infiltration, the mean value was 393 cells/mm^2^ (0–3035). Mean values of 1000 cells/mm^2^ (0–4833) for CD8^+^ stroma infiltration and 257 cells/mm^2^ (0–2269) for CD8^+^ tumor infiltration were determined. It was not possible to estimate a quantification in the tumor core. Only thresholds that showed significant differences in survival are mentioned in the following text. 

### 2.2. T-Cell Infiltration in Stroma

**Figure 1:**
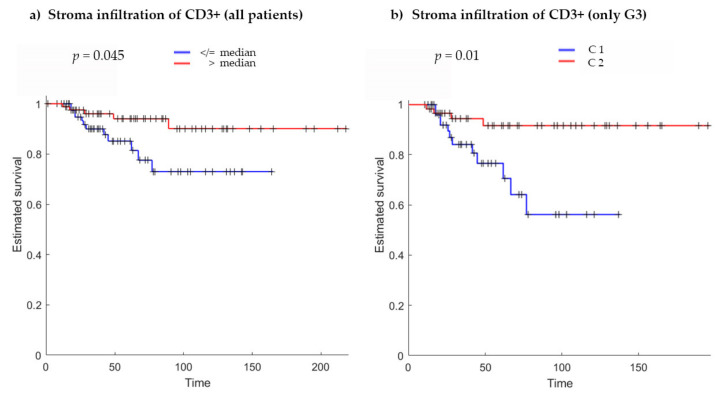
Kaplan–Meier curves for (**a**) stromal infiltration of CD3^+^ immune cells (all patients) using the median (2882 cells/mm^2^) as threshold to divide patients into two groups (*p* = 0.045). (**b**) Stromal infiltration of CD3^+^ immune cells (only in G3 subgroup) using the *k*-means clustering approach (*p* = 0.01). C1 with low and C2 with high immune cell infiltration. In each graph, Kaplan–Meier curves present a significant difference between the two groups, showing an increased survival in patients with higher immune cell infiltration. *p*-Values are calculated using the log-rank test; *x*-axis, survival in months; *y*-axis, estimated survival (fraction of individuals).

Patients were divided into two groups according to their stroma T-cell infiltration value by using various threshold approaches. By considering the 50th percentile (median) of the infiltrating immune cells in stroma as the cut-off value (2882 cells/mm^2^) and comparing the two groups using the Kaplan–Meier approach, a significant difference in survival time was found (*p* = 0.045), resulting in nearly 20% higher survival after 160 days for patients with a higher number of infiltrating immune cells in the stroma (Figure 1a).

For further analysis, patients were divided into two groups based on grading, G2 (*n* = 52) and G3 (*n* = 115). Comparing the two groups, no statistical difference was found for stroma T-cell infiltration. There were no pT1G1 tumors in the cohort (due to definition of pT1 BC).

Given the higher percentage of recurrence and progression to muscle-invasive disease within the G3 group, we decided to perform subgroup analyses on patients with G3 grading using the *k*-means clustering approach. The clusters identified with *k* = 2 (C1, *n* = 55, low stroma T-cell infiltration; C2, *n* = 60, high stroma T-cell infiltration) showed statistically significant different Kaplan–Meier curves (Figure 1b). A higher stroma T-cell infiltration showed better survival probability (*p* = 0.01). Interestingly, the minimum stroma infiltration value for cluster C2 coincided with the median of the infiltrating immune cells in stroma calculated on the whole dataset.

Furthermore, by using the chi-squared test, patients in the cluster with higher immune response showed significantly less recurrence (*p* = 0.034) and better overall survival (*p* = 0.019). 

No significant results were found for stroma infiltration with CD8^+^ T cells using the same analytical approaches as for CD3. It was not possible to find a cut-off value considering varied threshold approaches. When comparing the G2 and G3 subgroups, no differences were found for CD8^+^ stroma T-cell infiltration. There were no differences found for the G3 group separately using the *k*-means cluster analysis. 

### 2.3. T-Cell Infiltration in Tumor

When the same analysis was performed after setting thresholds on tumor T-cell infiltration values in CD3^+^ T cells and CD8^+^ T cells to separate patients and comparing the corresponding Kaplan–Meier curves, no differences were identified (Figure S1). 

**Figure 2:**
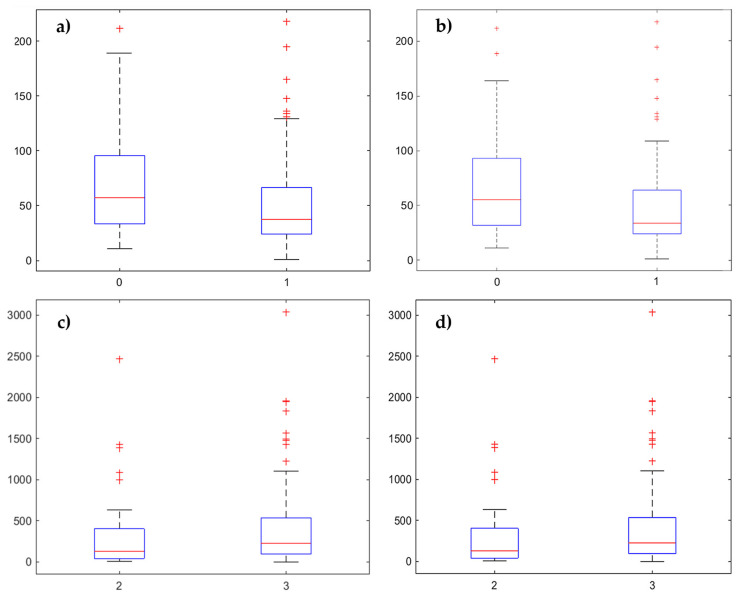
Box plots (**a**,**b**) comparing the median of tumor T-cell infiltration by using different percentiles to separate patients into two groups. Only the lowest *p*-values are shown in this composite figure. (**a**) CD3^+^ immune cell infiltration grouped by the 45th percentile (cut-off: 176 cells/mm^2^) (*p* = 0.011). (**b**) CD8^+^ immune cell infiltration grouped by the 50th percentile (cut-off: 118 cells/mm^2^) (*p* = 0.014). Box plots comparing (**c**,**d**) the median of tumor T-cell infiltration by using G2 versus G3 to separate patients into two groups. (**c**) CD3^+^ immune cell infiltration (*p* = 0.043) and (**d**) CD8^+^ immune cell infiltration (*p* = 0.028).

However, when considering the 45th percentile (cut-off: 176 cells/mm^2^) and the 50th percentile (cut-off: 210 cells/mm^2^) of tumor infiltration in CD3^+^ T cells as thresholds and comparing the median of the corresponding survival time distribution by means of the Wilcoxon rank sum test, an inverse trend compared with stroma T-cell infiltration was identified (45th percentile, *p* = 0.011; 50th percentile, *p* = 0.047) (Figure 2a and Figure S2a). A higher tumor T-cell infiltration at the invasive margin seemed to be associated with worse OS rates. All other 5th percentiles failed to separate patients into groups with significant differences.

Similar results were found for tumor T-cell infiltration of CD8^+^ immune cells considering the 40th percentile (cut-off: 84 cells/mm^2^, *p* = 0.045), the 45th percentile (cut-off: 102 cells/mm^2^, *p* = 0.017), the 50th percentile (cut-off: 118 cells/mm^2^, *p* = 0.014) and the 55th percentile (cut-off: 137 cells/mm^2^, *p* = 0.014) as thresholds (Figure 2b and Figure S2b–d).

Considering the classification of patients in the G2 and G3 groups, significant higher values for CD3^+^ (*p* = 0.043) and CD8^+^ (*p* = 0.028) tumor T-cell infiltration were found in the G3 group (Figure 2c,d).

Regarding the analysis of the abovementioned clinicopathological data with G3 subgroup showing a worse prognosis, the results seemed coherent, suggesting that a higher immune response in the tumor compartment at the invasive margin is associated with poor prognosis.

### 2.4. Tertiary Lymphoid Structures

**Figure 3:**
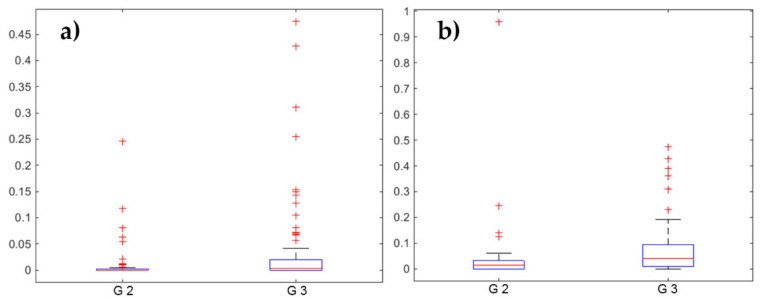
Box plots (**a**,**b**) comparing the numbers of (**a**) lymphocyte aggregates (LA) and (**b**) tertiary lymphoid structures (TLS) per mm^2^ when separating the patients into two groups by using G2 versus G3 subgroup in CD3^+^ stained slides. Significant higher values of LA (*p* = 0.0014) and TLS (*p* = 0.0003) were found in the G3 subgroup. Please note the different scaling of the graphs.

As a measure of the expression of immune reaction, we quantified tertiary lymphoid structures (TLS) and lymphocyte aggregates (LA) within the CD3^+^ stained slides. There was no difference between CD3^+^ and CD8^+^ in terms of TLS and LA presence. No significant differences were found in terms of survival comparing groups using several thresholds on TLS and LA values. However, when considering the classification of patients into G2 and G3 groups, significant higher values for LA (*p* = 0.0014) and TLS (*p* = 0.0003) were found in the G3 group (Figure 3).

### 2.5. Clinicopathological Data and Survival Endpoints

To identify prognostic markers, we analyzed the associations between tumor characteristics and various endpoints. Results are shown in Table S1. Statistically significant differences for recurrence-free survival (RFS), progression-free survival (PFS) and cancer-specific survival (CSS) were found by comparing G2 tumors with G3 tumors (grading according to the World Health Organization (WHO), 1973). The corresponding Kaplan–Meier curves likewise showed significantly better CSS for patients with G2 tumors (*p* = 0.022) (Figure S3a). Furthermore, local infiltrative growth pattern was associated with worse CSS (*p* = 0.032) (Figure S3b).

## 3. Discussion

The salient finding of the present study was that immune cell infiltration is associated with prognosis of T1 bladder cancer. Importantly, the exact localization of the immune cells matters. While CD3^+^ T cells in the stroma were associated with better prognosis, T cells—especially CD8^+^ cytotoxic T cells—in the tumor invasive margin seemed to be associated with worse outcomes. In our study cohort, more than 10% of the patients died due to their pT1 BC.

There are different subtypes of immune cells in the microenvironment of the tumor [14]. For instance, Bergmann et al. showed that less methylation of DNA sections that prime CD4^+^ Th1 cells results in better survival probability [15]. Shi et al. showed that a higher CD3^+^/CD4^+^ ratio predicts better survival in MIBC [16]. 

We decided to investigate CD3^+^ and CD8^+^ T cells as recommended by a published consensus-based advice on how to use the immunoscore [9] and because there are several studies which assume that these subtypes are of special prognostic value. Different studies diverge with respect to the predictive value of immune cells in different subcompartments. The first study published on this topic in 2007 recognized that intratumoral CD8^+^ T cells in MIBC were associated with better OS and DFS [17]. Subsequent studies arrived at corresponding findings. Otto et al. constituted intratumoral CD3^+^ T cells as a positive prognostic marker in NMIBC [18]. Ingels et al. and Pfannstiel et al. found that high numbers of intratumoral CD3^+^ and CD8^+^ T cells were associated with favorable outcomes in MIBC [10,19]. Despite these data, Lipponen et al. and Wang et al. showed that a higher number of CD8^+^ tumor-infiltrating lymphocytes correlated with poor OS [20,21]. Our results seemed consistent with these findings, showing that a higher number of immune cells in the tumor compartment is a negative predictive factor.

Conflicting results could be due to different inclusion criteria, various analyzed locations (invasive margin versus tumor core) and different material, ranging from naive TURB material of T1 tumors [18] to cystectomy tissue from T4 tumors [17]. Our study included exclusively naive tissue from stage pT1 BC, resulting in a more homogenous cohort without distortion due to early cystectomy or former immune therapy.

The aim of the worldwide immunoscore task force is to coordinate quantification efforts to be able to compare different findings and to reach harmonization and simplicity in implementation [8]. A current study by Yu et al. aimed to find a method for applying immunoscore to MIBC. The results seemed consistent with pre-existing findings in colorectal cancer, showing that a high immunoscore is associated with better DFS and OS [22].

The area of interest for stroma infiltration is directly adjacent to the invasive margin of the tumor. We analyzed this area with CD3^+^ T cells showing a positive impact on prognosis.

The second area of interest is the center of the tumor. Our samples often lacked a coherent tumor because of multifocality and fragmented tissues. In consequence, quantification in the tumor core was not possible, but only in the region adjacent to the invasive margin. Not only the appearance of CD3^+^ T cells, but specialized CD8^+^ T cells in particular, in the region adjacent to the invasive margin seemed to have a negative impact on prognosis. Further studies with larger case numbers are required to perform quantification in this region and, in addition, in the center of the tumor. Different ways to quantify the cells at the tumor core should be evaluated. These analyses could clarify the role of immune cells within the tumor, especially the importance of localization.

Tertiary lymphatic structures indicate an immune response in the tissue. Our findings showed that a higher number of TLS appeared within pT1G3 tumors and therefore, could be a negative predictive factor. In our study, we only saw a trend towards better prognosis. Additional studies are needed to refine these observations. 

Limitations of our study were due to the semiautomatic cell count. The program was not able to separate single cells in slides with high immune infiltration. We distinguished these areas by expert judgment. This method implied subjective selection. As there was a very high cell count, these patients belonged to the group with high immune infiltration in any case. Therefore, results of our study were not distorted. Another limitation of this study was its retrospective selection. Multiple margins of the tumor invasion appeared because of tumor multifocality. We analyzed three different non-contiguous regions with the highest immune reaction to minimize sampling error. 

According to the concept of immunoscore, we found CD3^IM^ as an independent prognostic marker for OS, showing higher values associated with better survival and trends according to the other sections (CD3^CT^, CD8^CT^, but not for CD8^IM^) especially in the G3 group. This group is under focus, because pT1G3 bladder cancer often appears as an aggressive tumor with a high tendency to progress into muscle-invasive disease. Future studies should investigate whether quantification of immune response could be used in clinical decision making and bring us closer to more individualized treatment. The controversial issue of which therapy is best for each individual—a bladder-sparing approach versus early cystectomy or in some other words, overtreatment versus delayed cystectomy—remains unresolved. In addition, new prognostic markers could be used to identify patients taking benefit from closer follow-up examinations. Adjustments in patient stratification could help in earlier detection of tumor progression. A lack of independent markers causes pT1G3 bladder cancer to be one of the most challenging malignancies in urology. 

## 4. Materials and Methods

### 4.1. Clinicopathological Data

The study included tissues from untreated patients who were first diagnosed with stage pT1 bladder cancer and treated by TURB without early cystectomy between 1989 and 2009 at the Department of Urology, St. Josef Medical Centre, Regensburg University. Clinical management according to the European Association of Urology (EAU)guidelines was performed in an outpatient setting [2]. A second TURB was carried out 4–6 weeks later. Progression into muscle-invasive disease prompted radical cystectomy. These patients were excluded from the study (*n* = 12). In addition, 19 patients were screened, but excluded from the analysis because too little material was obtained to meet study criteria (three independent regions for analysis per patient). Of the remaining 167 patients, 18 underwent secondary cystectomy and 149 patients were treated by the organ-preserving approach. Eighty-two patients received instillations with bacillus Calmette–Guérin (*n* = 56) or mitomycin C (*n* = 26).

The median age at diagnosis was 72 years (range: 42–98 years) with a male-to-female ratio of 4:1. This ratio was higher than the normal distribution, with men being three times more frequently affected by urothelial carcinoma than women [23]. 

Pathological characteristics were assessed by two experienced uropathologists (A.H. and S.B.).

Endpoints were PFS defined as time to recurrence with tumor disease higher than stage pT1 (showing at minimum, an invasion into lamina muscularis propria), CSS and OS. Twenty-six (15.6%) patients showed progression. Seventeen (10.2%) patients died due to advanced bladder cancer and fifty-nine patients (35.3%) died due to diseases other than BC.

The median follow-up period (range: 1–218 months) was 43 months.

### 4.2. Immunhistochemistry and Quantification

Whole slide sections were stained for CD3 (Zytomed, Berlin, Germany; monoclonal rabbit; dilution 1:150) and CD8 (Dako, Carpinteria, CA, USA; monoclonal murine; dilution 1:100) with clinical-grade antibodies according to manufacturer’s instructions using the Ventana Benchmark Ultra autostainer (Ventana, Tucson, AZ, USA). The bound primary antibodies were detected with the visualization reagent linked to a dextran polymer backbone with DAB (3,3-diaminobenzidine) as a chromogen solution. Afterwards, the sections were counterstained with Meyer’s hematoxylin. 

### 4.3. Digital Image Analysis and Region of Interest (ROI) Selection

**Figure 4:**
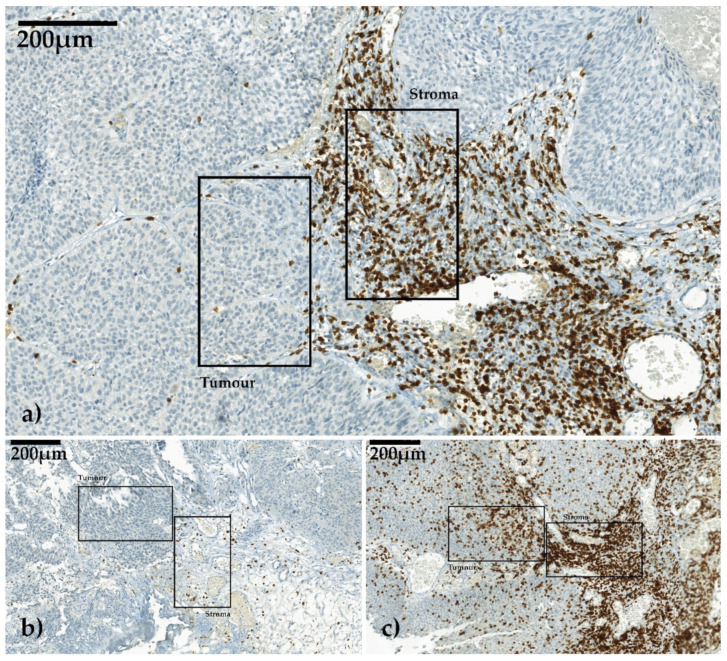
Three different CD3-stained slides in non-muscle-invasive bladder cancer (NMIBC) viewed in Panoramic Viewer 1.15.4. Selection of one high-power field (HPF) each for stroma and for tumor infiltration. Invasion front was between these two corresponding HPFs. The images were scanned with digital slide scanner (camera: CIS VCC, Panoramic MIDI Scan; 3DHistech Ltd., Budapest, Hungary). (**a**) Example for moderate stroma infiltration and low tumor infiltration, (**b**) example for low stroma infiltration and low tumor infiltration, (**c**) example for high stroma infiltration and high tumor infiltration.

All immunohistochemically stained slides were scanned with a digital slide scanner (camera: CIS VCC, Panoramic MIDI Scan; 3DHistech Ltd., Budapest, Hungary) at a magnification of 1:400, transferred to workstation (resolution 0.11 µm/pixel) and examined in Panoramic Viewer (Vers. 1.15.4 3DHistech Ltd., Budapest, Hungary). We determined three non-contiguous areas that showed the highest immune response by infiltrating lymphocytes at the invasive margin as the region of interest (ROI) and best fit for the analysis. In each ROI, two high-power fields (HPFs) of 0.087 mm^2^ each, one in the stromal compartment and one in the tumor, both adjacent to the invasive margin, were cut out digitally (Figure 4a–c). 

**Figure 5:**
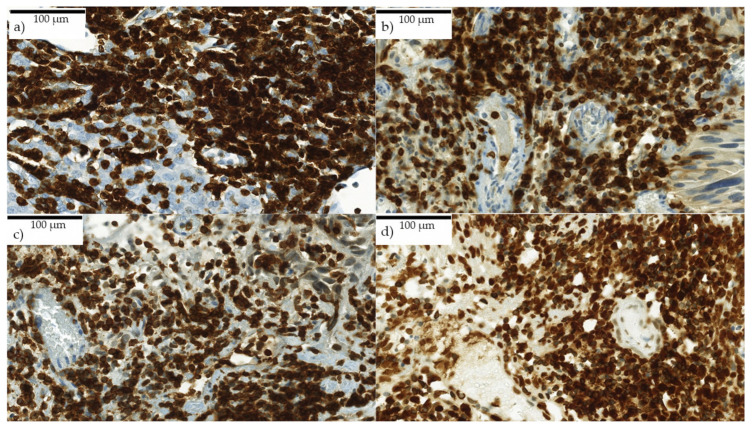
(**a**–**d**) Very high immune reaction in CD3^+^ stained slides. Each picture shows one HPF in the stroma. Cells were displayed superimposed. Exact differentiation of single cells was not possible for the program. Scanned with digital slide scanner (camera: CIS VCC, Panoramic MIDI Scan; 3DHistech Ltd., Budapest, Hungary).

CD3^+^ and CD8^+^ T cells were counted using the semiautomatic image analysis software COUNT (Biomas Vers. 1.0, L.D., Erlangen, Germany), that recognizes immune cells automatically with a high specificity. With the subsequent visual adjustment, a high sensitivity was achieved. Areas with high density of lymphocytes in which it was not possible to identify single cells automatically were summarized as one area manually and subsequently divided into circles with 57 µm perimeter by the program. For clusters with an extremely high density of lymphocytes, dividing in 45 µm perimeter was used, because cells were displayed superimposed on other cells (Figure 5a–d). The number of labelled cells was determined per cells/mm^2^. 

**Figure 6:**
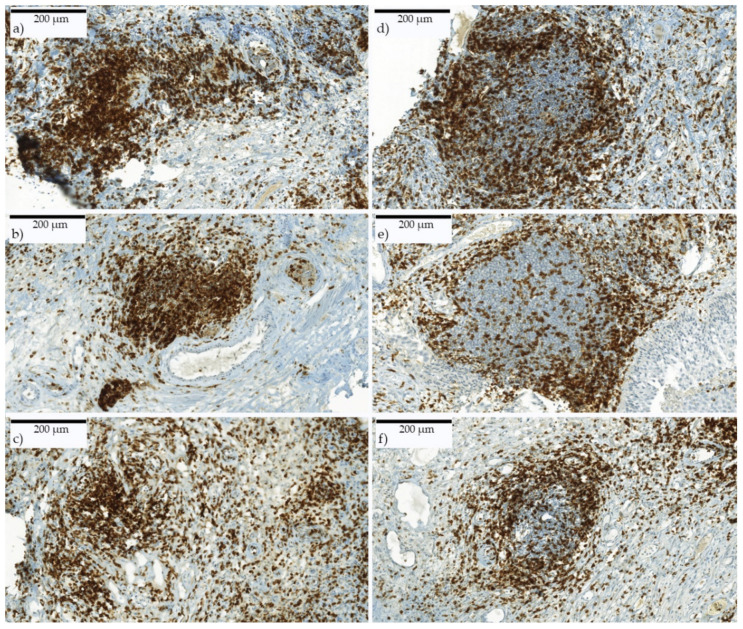
(**a**–**c**) Lymphocyte aggregates and (**d**–**f**) tertiary lymphoid structures. CD3^+^ immune-stained slides. LA consist of clustered immune cells. TLS show organized structures such as an active center with surrounding immune cells. Scanned with digital slide scanner (camera: CIS VCC, Panoramic MIDI Scan; 3DHistech Ltd., Budapest, Hungary).

Furthermore, the total number of LA and TLS in whole slides was detected and put in relation to the tissue size. LA were defined to an extent of clustered immune cells with an area of at least 0.0435 mm^2^ (complied to 0.5 HPF) (Figure 6a–c). TLS were identified by their typical appearance including an active germinal center (Figure 6d–f). The reviewer was blinded for all patients’ clinical data. 

### 4.4. Statistics 

Statistical analyses were performed using MATLAB v. R2014b (The MathWorks, Inc., Natick, MA, USA). 

The association of tumor characteristics with cancer-specific survival, recurrence and progression was tested by means of chi-squared test. In case of significant association (*p*-value < 0.05), Kaplan–Meier curves were evaluated. 

Based on infiltrating immune cells in stroma and tumor, several threshold strategies including subintervals, 5th percentiles and mean value were used to distinguish patients into two groups. Kaplan–Meier curves were estimated and statistically compared using the log rank test. 

*k*-Means clustering was applied using stroma T-cell infiltration as the feature, the squared Euclidean distance as the measure and the *k*-means++ algorithm for cluster center initialization.

## 5. Conclusions

Our study demonstrated that quantification of immune response is a promising approach for identifying patients with pT1 bladder cancer showing a high progression risk and poor prognosis. Prognostic markers enable uro-oncologists to optimize patient management, whether through closer follow-up examinations or personalized treatment. Further studies with increased numbers of cases and defined strategies in quantification could help to establish a standardized immunoscore in clinicopathological routines. 

## Supplementary Materials

The following are available online at https://www.mdpi.com/1422-0067/21/21/8407/s1.

## Abbreviations

BC	bladder cancer
BCG	bacillus Calmette–Guérin
BRIDGE	Bladder Cancer Research Initiative for Drug Targets Germany
CSS	cancer-specific survival
DFS EAU	disease-free survival European Association of Urology
HPF	high power field
LA	lymphocyte aggregates
MD	medical doctor
MIBC	muscle-invasive bladder cancer
NMIBC	non-muscle-invasive bladder cancer
OS	overall survival
PFS	progression-free survival
RFS	recurrence-free survival
ROI	region of interest
TILs	tumor-infiltrating lymphocytes
TLS	tertiary lymphoid structures
TURB	transurethral resection of the bladder
WHO	World Health Organization
