# Supplementary material for: High Stroma T-Cell Infiltration is Associated with Better Survival in Stage pT1 Bladder Cancer
Source: Int J Mol Sci. 2020 Nov 9;21(21):8407. doi: 10.3390/ijms21218407 (PMC7665154; doi:10.3390/ijms21218407)
Supplement: Supplementary file 1 [file ijms-21-08407-s001.zip › ijms-948195-supplementary.docx]

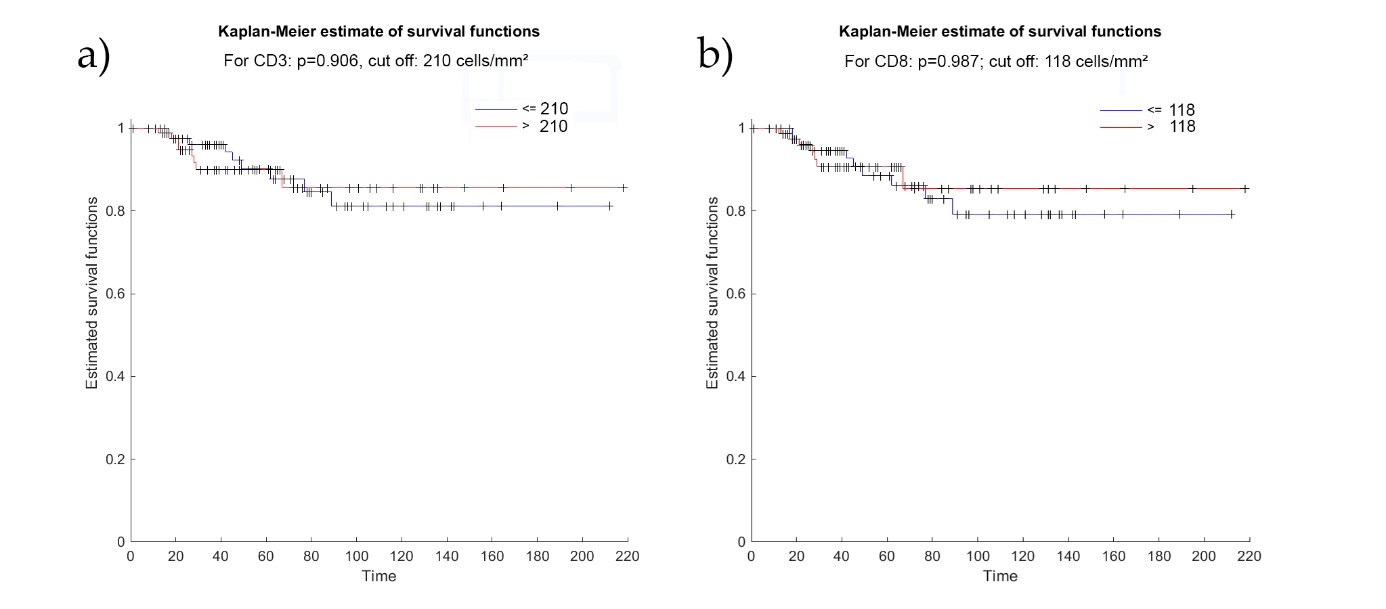


**Figure S1.** Examples of non-significant Kaplan-Meier curves for (a) CD3+ tumour T cell infiltration; p=0.906, cut off: 210 cells/mm² and (b) CD8+ tumour T cell infiltrations; p=0.987, cut off: 118 cells/mm². No significant results were found for tumour infiltration using varied threshold approaches. X: survival in months (max. 220 months), Y: percentage of individuals (max. 100%).


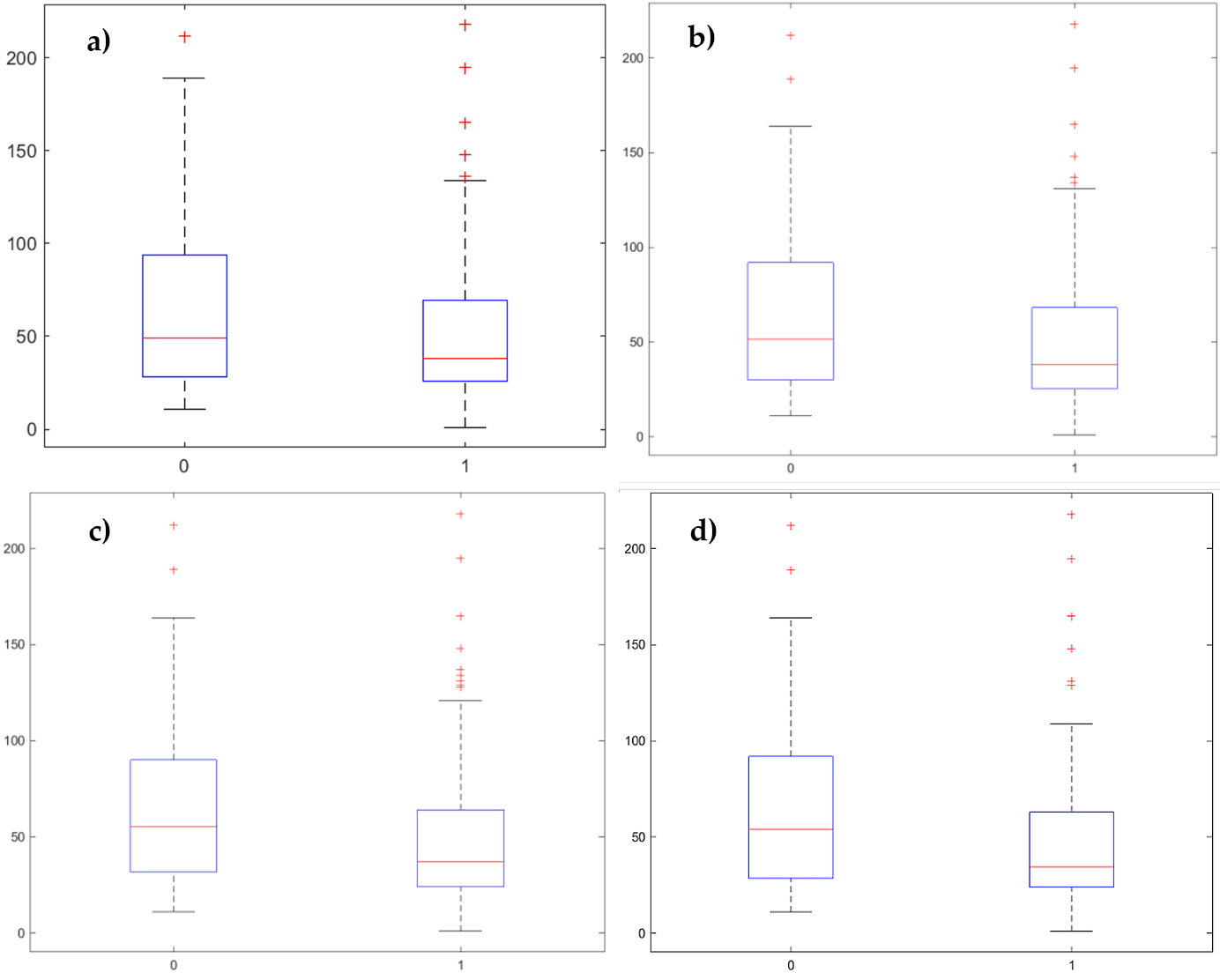


**Figure S2.** Box plots comparing the median of tumour T-cell infiltration by using different percentiles to separate patients into two groups. Lowest p-values are shown in the fluent text in Figure 2. This composite figure shows the remaining box plots. (a) CD3+ immune cell infiltration grouped by the 50th percentile (cut-off: 210 cells/mm²) (p=0.047) (b) CD8+ immune cell infiltration grouped by the 40th percentile (cut-off: 84 cells/mm²) (p=0.045) (c) CD8+ immune cell infiltration grouped by the 45th percentile (cut-off: 102 cells/mm²) (p=0.017) (d) CD8+ immune cell infiltration grouped by the 55th percentile (cut-off: 137 cells/mm²) (p=0.014)

|  | total | RFS | | P-val | PFS | | P-val | CSS | | P-val |
| --- | --- | --- | --- | --- | --- | --- | --- | --- | --- | --- |
|  |  | num | *% of total* |  | num | *% of total* |  | num | % of total |  |
| Grading WHO 1973  G2  G3 | 52  115 | 7  37 | *13.5*  *32.2* | **0.0110** | 3  23 | *5.8*  *20* | **0.0188** | 1  16 | 1.9  13.9 | **0.0177** |
| Multifocality  Unifocal  Multifocal | 136  31 | 38  6 | *27.9*  *19.4* | 0.3274 | 23  3 | *16.9*  *9.7* | 0.3161 | 16  1 | *11.8*  *3.2* | 0.1559 |
| Carcinoma in situ  Yes  No | 41  126 | 13  31 | *31.1*  *24.6* | 0.3697 | 9  17 | *22*  *13.5* | 0.1944 | 7  10 | *17.1*  *7.9* | 0.0928 |
| Tumor size  <3cm  >3cm | 76  91 | 16  28 | *21.1*  *30.8* | 0.1558 | 11  15 | *14.5*  *16.5* | 0.7213 | 5  12 | *6.6*  *13.2* | 0.1596 |
| Growth  Papillar  Solid | 150  17 | 41  3 | *27.3*  *17.7* | 0.3902 | 25  1 | *16.7*  *5.9* | 0.2451 | 15  2 | *10*  *11.8* | 0.8196 |
| Invasion pattern  non-infiltrativ  infiltrative | 131  36 | 33  11 | *25.2*  *30.6* | 0.5175 | 19  7 | *14.5*  *19.4* | 0.4690 | 10  7 | *7.6*  *19.4* | **0.0379** |
| Instillation therapy  Mitomycin C  Bacillus Calmette Guerin | 26  56 | 4  14 | *15.4*  *25* | 0.3276 | 3  7 | *11.5*  *12.5* | 0.9015 | 1  3 | *3.9*  *5.4* | 0.7676 |

**Table S1.** Clinicopathological data. Associations of tumour characteristics with RFS, PFS and CSS. WHO-Grading and invasion pattern show significant different survival probabilities.


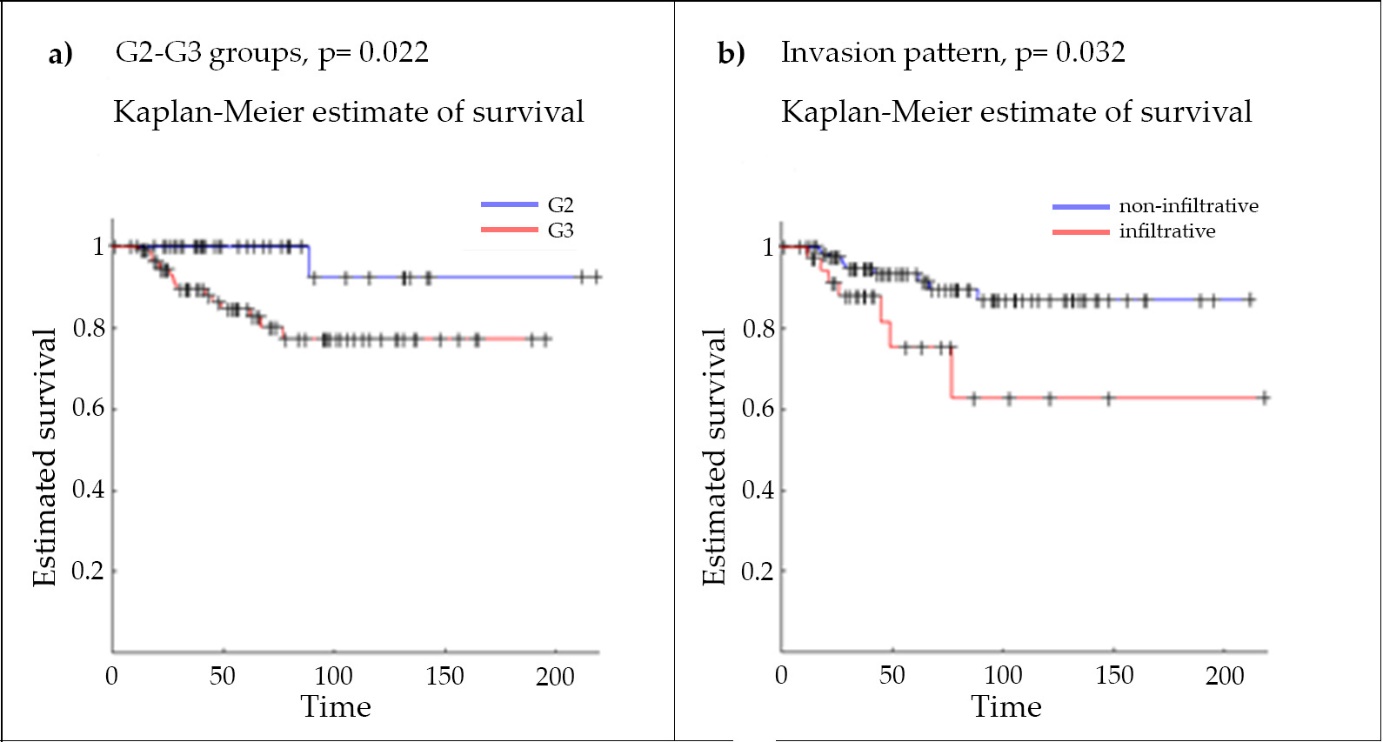


**Figure S3.** Kaplan-Meier curves for (a) WHO-Grading 1973 and (b) infiltrative growth. X: survival in months (max. 240 months), Y: percentage of individuals (max. 100%). The graphs show an increased survival in patients with G2-grading (p=0.022) and non-infiltrative growth (p=0.032).
